# A nonenzymatic reduced graphene oxide-based nanosensor for parathion

**DOI:** 10.3762/bjnano.13.65

**Published:** 2022-07-28

**Authors:** Sarani Sen, Anurag Roy, Ambarish Sanyal, Parukuttyamma Sujatha Devi

**Affiliations:** 1 Functional Materials and Devices Division, CSIR-Central Glass & Ceramic Research Institute, 196 Raja S.C. Mullick Road, Jadavpur, Kolkata 700032, India; 2 National Institute of Technology Durgapur, Mahatma Gandhi Road, A-Zone, Durgapur, West Bengal 713209, India; 3 Environment and Sustainability Institute, University of Exeter, Penryn Campus, Cornwall TR10 9FE, United Kingdom; 4 Chemical Sciences and Technology Division, CSIR-National Institute of Interdisciplinary Science and Technology, Thiruvananthapuram, Kerala 695019, India

**Keywords:** electrochemical nanosensor, graphene oxide, nonenzymatic approach, parathion, pesticides, square-wave voltammetry

## Abstract

Organophosphate-based pesticides (e.g., parathion (PT)) have toxic effects on human health through their residues. Therefore, cost-effective and rapid detection strategies need to be developed to ensure the consuming food is free of any organophosphate-residue. This work proposed the fabrication of a robust, nonenzymatic electrochemical-sensing electrode modified with electrochemically reduced graphene oxide (ERGO) to detect PT residues in environmental samples (e.g., soil, water) as well as in vegetables and cereals. The ERGO sensor shows a significantly affected electrocatalytic reduction peak at −0.58 V (vs Ag/AgCl) for rapid quantification of PT due to the amplified electroactive surface area of the modified electrode. At optimized experimental conditions, square-wave voltammetric analysis exhibits higher sensitivity (50.5 μA·μM^−1^·cm^−2^), excellent selectivity, excellent stability (≈180 days), good reproducibility, and repeatability for interference-free detection of PT residues in actual samples. This electrochemical nanosensor is suitable for point-of-care detection of PT in a wide dynamic range of 3 × 10^−11^–11 × 10^−6^ M with a lower detection limit of 10.9 pM. The performance of the nanosensor was validated by adding PT to natural samples and comparing the data via absorption spectroscopy. PT detection results encourage the design of easy-to-use nanosensor-based analytical tools for rapidly monitoring other environmental samples.

## Introduction

Crop production is constantly increasing to fulfil the demands of the growing population. The protection of crops against insects is a big challenge for our society. Pesticides have indiscriminately been used in all sectors of agriculture. Among the various pesticides available for the aforementioned purpose, organophosphates (OPs) are commonly employed in agriculture, households, gardens, and veterinary practices. This practice also jeopardizes food safety in all stages of the food supply chain, even after pesticide use. Due to its high nondegradability, pesticides can stay more often on the surface of fruits and vegetables; sometimes, it can also penetrate into the peel of vegetables and fruits [1]. Organophosphorus insecticides react with biomolecules either via deoxyribonucleic acid (DNA) alkylation or acetylcholinesterase (AChE) phosphorylation, involved in the initiation of the carcinogenic process and acute cholinergic toxicity, respectively [2]. Parathion (PT) is a highly toxic OP-based insecticide, potentially harmful to human health, and it may even cause death upon ingestion, inhalation, or dermal penetration [3–4]. Due to its extreme toxicity, it is necessary that easy-to-use, cost-effective diagnostic kits for routine screening of pesticides in fruits and vegetables are developed.

The high-throughput analytical methods such as chromatographic (gas, liquid) and spectroscopic (mass, absorption, fluorescence) techniques are time-consuming, laborious, costy, require specific and sophisticated instruments and trained personnel, and most often are not portable to enable on-site detection [5–6]. Electrochemical nanosensors are one of the preferred methodologies due of their fast and straightforward responsive nature, high sensitivity, and selectivity leading to real-time detection [7]. A combination of a receptor, an analyte, and a transducer is made up to obtain an electrochemical sensor, in which the surface of the electrode induces redox characteristics via selective binding with the analyte under a voltage for a different analyte which results in a quick qualitative signal. This approach promotes real-time label-free methods, providing more consistent and reproducible results.

Most of the electrochemical nanobiosensors for the detection of OPs (e.g., methyl parathion, ethyl parathion, fenitrothion, chlorpyrifos, paraoxon, ethion, and acephate) are based on the inhibition of acetylcholinesterase (AChE) activity (an indirect method) [8–10]. Some organic molecules and metal cations also act as an inhibitor of AChE. Thus, interference-free detection of OP in agricultural samples using enzyme-based nanosensors is challenging. The stability of bionanosensors also extensively depends on the viability of the corresponding biomolecule during the matrix immobilization course [9]. The main drawbacks of using bionanosensors for the selective detection of OP in actual samples are i) the high cost of the enzyme, ii) low stability of biomolecules at room temperature, and iii) difficulties in using interference-free selective detection of a specific OP. However, nonenzymatic electrochemical nanosensors could easily be employed as rapid, cost-effective, easy-to-handle, selective, sensitive, and point-of-care (POC) analytical tools for monitoring environmental pollutants [2,11]. They can also detect residual OPs based on their electrocatalytic activity and affinity toward nanomaterials, such as nanoparticles, carbon nanomaterials, and metal oxides [11]. In a few reports, hybrid carbon nanomaterials such as ferrocene-thiophene modified by carbon nanotubes, zinc(II) phthalocyanine-boron dipyrromethene attached single-walled carbon nanotubes were used for the direct detection of pesticides [12–15]. So far, only limited electrochemical nanosensors modified by nanomaterials have been reported to detect PT [16–17]. However, their sensitivity and detection limit for quantifying trace amounts of PT in environmental samples are improved. The inherent electrochemical behavior of nitroaromatic OPs (e.g., paraoxon, parathion, and fenitrothion) exhibit well-defined redox activities at the electrode surface, potentially leading to the fabrication of nonenzymatic electrochemical nanosensors for detecting specific OPs on the electroactive surface [2,11,17–19]. For example, electrochemical sensing platforms modified with zirconia-embedded PEDOT membrane, graphene nanoribbons doped with silver nanoparticles, rGO doped with ZrO_2_, and CuO–TiO_2_ hybrid nanocomposites were proposed to detect methyl parathion [19–22]. Rajaji et al. (2019) modified glassy carbon electrodes with graphene oxide encapsulated 3D porous chalcopyrite (CuFeS_2_) nanocomposites to detect methyl paraoxon in vegetables [23]. Recently, Jangid et al. (2021) also described the electrocatalytic activity of fenitrothion on glassy carbon electrodes modified with nitrogen and sulfur co-doped activated carbon-coated multiwalled carbon nanotubes [24]. Nevertheless, the fabrication process of the sensing platform was not cost-effective, stable, and sensitive in order to develop a robust electrochemical nanosensor for on-site monitoring of organophosphates in agricultural samples. To date, no reliable sensing system is available for the rapid quantification of parathion residues in environmental samples. Thus, the primary goal of this report was to showcase the fabrication of a more effective, economical, electroactive surface in a simplified way to selectively detect PT residues in real samples. Thus, a robust sensing matrix can be used for designing a nonenzymatic POC device with a low detection limit and long-term stability at room temperature.

Graphene oxide (GO), consisting of a monolayer of sp^2^-hybridized carbon atom network, has already been used in electrocatalysis, nanoelectronics, bionanosensors, and sustainable energy storage systems due to its larger active surface area, enhanced electron transport facility, excellent mechanical, thermal, and electrical stability [11,25–27]. The electronic structure and surface physicochemistry of graphene are beneficial for electron transfer. Several graphene-based nanocomposites based on complex synthesis processes are reported as excellent sensing matrices for detecting various analytes [11,21–23,28]. The "green synthesis" of graphene via electrochemical reduction is the most economical strategy for the mass production of graphene compared to chemical or thermal reduction of GO [27,29]. Since no hazardous chemicals (e.g., hydrazine) as reductants or rapid heat treatment at high temperatures are required for the synthesis of electrochemically reduced GO (ERGO), controlled synthesis of ERGO films could be possible via optimization of electrochemical parameters. These parameters are the range of the applied voltage, numbers of cycles, the scan rate of cyclic voltammetry, or reduction time at a fixed potential in chronoamperometry [30–32].

However, the desired size and thickness of the film can be increased by controlling the amount of precursor GO deposited onto the electrode surface [31–32]. Optimizing the process parameters is a robust scientific approach to achieving the highest sensing performance of an electroactive analyte.

In this work, we proposed a simple, robust, and reliable ERGO-modified nonenzymatic electrochemical nanosensor as a good alternative for a POC-based easy diagnostic platform to monitor the level of PT residues in environmental samples, such as water, soil, crops, and vegetables. A straightforward and economic fabrication process with high sensitivity, selectivity, and stability with the lowest detection limit is the foremost advantage emerging from this study for the rapid on-site monitoring of PT. For this purpose, the electrochemical reduction of GO was tuned using various electrolytic buffers with different pH values, supporting the variation of the physicochemical characteristics of ERGO discussed in this work. Besides, this study highlights the scope of an interference-free nonenzymatic approach through electrochemical nanosensing which can also be used in other biosensing applications.

## Experimental

### Chemicals

The chemicals used in this work are summarized in Supporting Information File 1, Table S1. Sodium dihydrogen phosphate was used to prepare phosphate buffer saline (pH 4.6, 7.4, 9). Acetate buffer (20 mM, pH 4.5), and Britton–Robinson (BR) buffer (40 mM, pH 4) consisting of phosphoric acid, boric acid, and acetic acid were also prepared.

### Synthesis of graphene oxide

Graphene oxide was synthesized from graphite powder using a modified Hummer’s method [30–31]. In detail, 100 mg of sodium nitrate (Merck) was added to 250 mg of graphite powder (Alfa Aesar) and further acidified with ≈5 mL of concentrated sulfuric acid (Merck) at a temperature range of 0–5 °C followed by vigorous stirring. In the next step, 600 mg of KMnO_4_ (Merck) was sequentially added to the aforementioned solution, during which the solution temperature was increased to 35 °C. After 30 min of the addition of KMnO_4_, a brownish-grey paste was obtained. Deionized water (100 mL) was added to the paste under constant stirring at 90 °C for 30 min, followed by dropwise addition of 30% H_2_O_2_ (Merck, India). Finally, a dark brown solution was filtered and thoroughly washed with 100 mL of distilled water until a neutral pH value was achieved. The black product obtained after filtration was dispersed in water and sonicated for 1 h to get a well-dispersed suspension. Finally, the suspension was centrifuged twice at 3000 rpm for 15 min. The product (GO) was collected and dried at room temperature for further studies.

### Fabrication of electrochemically reduced graphene oxide modified electrodes

Before surface modification of GO, a bare glassy carbon electrode (GCE, φ = 3 mm) was polished in 1.0, 0.3, and 0.05 micron alumina slurry (CHI Instruments) on micro cloth pads sequentially to a mirror-like finish with fine wet emery paper (grain size 4000), and rinsed with ultrapure water. Then the electrode was separately dipped into concentrated NaOH, nitric acid, and methanol for 120 s, followed by sonication in alcohol for 2 min, and finally dried in air. The as-prepared GO colloidal suspension (2 mg·mL^−1^) was deposited onto the surface of the pretreated GCE and dried at room temperature. The GO/GCE was submerged in 50 mM PBS, pH 4.6, for the electrochemical reduction of GO by a potentiostat technique at a potential of −0.9 V for 900 s using an Ag/AgCl reference electrode. The buffer and pH values of the electrolytes were optimized to fabricate electrochemically reduced GO (ERGO) modified GCE designated as ERGO/GCE.

Cyclic voltammogram (CV) measurements to assess the electrochemical behavior of parathion were performed from +0.5 to −1.0 V versus Ag/AgCl, with a scan rate of 100 mV·s^−1^. Square-wave voltammetry (SWV) analysis was performed from −0.3 to +0.9 V versus Ag/AgCl, with pulse amplitude of 100 mV, frequency of 25 Hz, and modulation time of 10 s in 50 mM PBS. The nanosensor was cycled 25 times for signal stabilization before PT detection.

### Preparation of environmental and food samples for residual parathion analysis

The practical application of the proposed electrochemical nanosensor was studied by sensing PT in the groundwater, soil, tomato, and rice samples with different concentrations of PT. The groundwater and soil were collected from local agricultural land in Kolkata, India. As parathion is highly soluble in alcoholic compounds, we have used ethanol to extract residual PT from the collected food and environmental samples. The soil sample (1 g) was stirred for 1 h in 50% ethanol to disperse all organic and inorganic soil molecules in the liquid phase. Tomato as a sample vegetable was purchased from the local market in Kolkata, India, and washed with running water before preparing the sample. The tomato samples (30 g) were smashed with 30 mL of 50% ethanol, and the juice was collected for further filtration. Boiled rice (20 g) was also smashed with 20 mL of 50% ethanol. All the samples were stored at 4 °C after filtration (pore size = 0.45 micron) to remove all the solid impurities. Actual samples were spiked with different concentrations of PT during electrochemical analysis. Each concentration of PT was tested five times, and the average value was represented with standard deviation. The results also validate the standard spectrophotometric analysis.

### Quantification of parathion using spectrophotometry

The ultraviolet–visible (UV–vis) absorption spectroscopic study was performed to validate the results of the proposed nanosensor. A stock solution (1 mM) of PT was prepared in 99.9% ethanol (Empura, Merck). A 5 mL volume of working solutions of 1 to 35 µM was prepared in ethanol for monitoring the UV spectra in the range of 200–400 nm with a scan rate of 2 nm/s. The absorbance change of PT due to π–π* transition was noted at 273 nm using ethanol as blank, and a calibration curve was plotted to compare the results obtained from the proposed nanosensor.

### Material characterization

Voltammetric studies were carried out using an IVIUMStat electrochemical analyzer (Model: A09050, Iviumstat Technologies, USA), which was connected by a three-electrode system, including a modified and/or unmodified GCE as the working electrode, a saturated Ag/AgCl as the reference electrode (RE), and a platinum wire as the counter electrode (CE). The electrochemical impedance spectroscopy (EIS) study of the modified electrodes was carried out in 5 mM of [Fe(CN)_6_]^3−^ and [Fe(CN)_6_]^4−^ with 0.1 M KCl within the frequency range from 1 MHz to 0.01 Hz, amplitude of 10 mV, at a fixed potential of 0.28 V.

The UV–visible absorbance spectra were obtained on a UV–vis–NIR spectrophotometer (SHIMADZU UV-3600). The Raman spectra of the samples were recorded in the 1000–3500 cm^−1^ region with a resolution of 1 cm^−1^ using a Renishaw via a Reflex micro-Raman spectrometer with an argon ion (514.6 nm) laser. The X-ray photoemission spectroscopy (XPS) data were obtained from a PHI 5000 Versa probe II scanning XPS microprobe (ULVAC-PHI, U.S.) with monochromatic Al Kα (hν = 1486.6 eV) radiation, and a beam size of 100 μm. The Fourier transform infrared (FTIR) absorption spectra of GO and ERGO were collected in the 4000–400 cm^−1^ region on a Perkin Elmer spectrometer as KBr (Sigma-Aldrich, Germany) pellets. The crystalline phase of GO and RGO was characterized by X-ray diffraction (XRD) using a X’pertpro MPD XRD (PAN analytical B.V., the Netherlands) with Cu Kα radiation (λ = 1.5406 Å).

Scanning electron microscopy (SEM) of the modified electrode was conducted on a JEOLEVO^®^ 18 special edition (model: ZEISS EVO-MA 10) at an acceleration voltage of 15 kV. The morphological characteristics of the electrodeposited ERGO were obtained by field-emission scanning electron microscopy (FESEM, model: LEO 430i, Carl Zeiss) and high-resolution transmission electron microscopy (model: Tecnai G2 30ST, FEI) operating at 300 kV.

## Results and Discussion

### Optimization and characterization of electrochemically reduced graphene oxide formation

**Figure 1:**
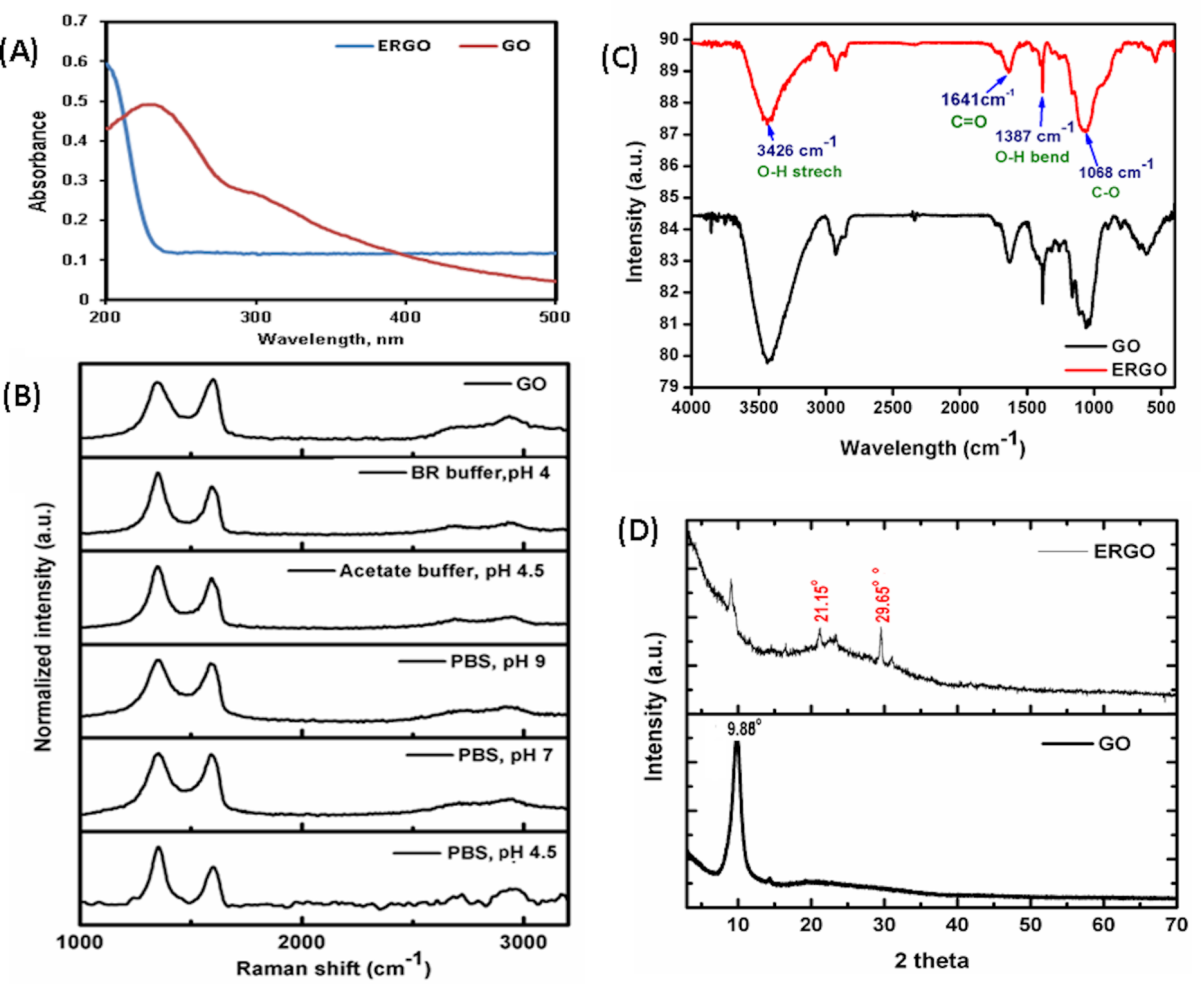
(A) UV–vis spectra of GO and ERGO. (B) Raman spectra for GO to ERGO conversion using different buffers as electrolytes. (C) FTIR spectra and (D) XRD patterns of GO and ERGO (PBS pH 4.5).

Figure 1A shows the UV spectra of GO and its change following the electrochemical reduction of GO. It is observed that the absorption peak of GO at 223 nm due to the π–π^*^ transition of the C=C bond disappeared in ERGO. The amount of residual oxygenated functional groups in ERGO films is likely to vary depending on the experimental conditions, such as applied potential, reduction times, and the electrolyte used [25]. The process parameters for electrochemical reduction were optimized to develop better functioning electrodes. Raman spectroscopy has been frequently used as a reliable technique to optimize the electrochemical parameters for the synthesis of ERGO in terms of the intensity ratio of D- (disordered band) to G-band (graphitic band) (*I*_D_/*I*_G_). It measures the change in size of the sp^2^ ring clusters in a network of sp^3^- and sp^2^-bonded carbon [33]. Previous reports have indicated the possibility of converting GO to ERGO at different electrochemical parameters, but its effect on the *I*_D_/*I*_G_ value have not been reported [25,29,33]. In this report, the pH value and buffer composition of the electrolyte were optimized to increase the deoxygenation of the GO sheet during ERGO formation. Figure 1B depicts three significant Raman peaks of GO at 1350 cm^−1^ for the D band (associated with defects in the sp^2^ lattice), 1596 cm^−1^ for the G band (due to vibrations of the hexagonal lattice), and 2700 cm^−1^ for the 2D band (related to numbers of layers in the graphene sheet). Table 1 shows the values of *I*_D_/*I*_G_ at different electrolytic buffers during one-step electroreduction of GO at a constant potential of −0.9 V. The intensity of *I*_D_/*I*_G_ predominantly increased for ERGO compared to the that of the as-prepared GO, which suggests a decrease in size of the sp^2^ domain due to extensive deoxygenation of the graphene sheets after electrochemical reduction. The comparative values of *I*_D_/*I*_G_ (Table 1) also indicate that a higher defect in the sp^2^ domain was observed at acidic pH values of the electrolytic buffer during electrochemical reduction of GO. The highest value of *I*_D_/*I*_G_ was found to be 1.454 for the conversion of ERGO using PBS (pH 4.5), which suggests the formation of higher defects between the graphene layers during electrochemical reduction [26,34]. Thus, 50 mM PBS, pH 4.5, has been chosen for an efficient conversion of GO to ERGO.

**Table 1:** Experimental sample table showing variation of Raman peak intensity ratio of ERGO using different electrolytes.

Sample in different pH	*I*_D_	*I*_G_	*I*_D_/*I*_G_	*I*_2D_	*I*_2D_/*I*_G_

GO	0.975524	1.01748	0.958765	0.43182	0.442654
acetate buffer, pH 4.5	1.00602	0.819277	1.227936	0.21328	0.212004
BR buffer, pH 4	1.01807	0.792169	1.285168	0.225525	0.221522
PBS buffer pH 4.5	1.00301	0.68976	1.454143	0.33042	0.329428
PBS buffer, pH 7	1.03313	0.978915	1.055383	0.29371	0.284291
PBS buffer, pH 9	1.03012	0.942771	1.092651	0.25699	0.249476

Figure 1C shows the characteristic FTIR spectra of GO and ERGO (in PBS, pH 4.5) to identify the change of functional groups due to electrolytic reduction of GO. The predominant characteristic absorption peaks of GO include a broad peak at 3426 cm^−1^ corresponding to the O–H stretching vibration originating from carboxyl groups. Besides, an intense peak at 1641 cm^−1^ was assigned to the C=O stretching of carboxyl and/or carbonyl groups, a sharp peak at 1387 cm^−1^ corresponding to a –OH bend, and a strong peak at 1068 cm^−1^ ascribed to an alkoxy and/or epoxy C–O stretching vibration. The significant reduction of the FTIR signal intensity of ERGO for –OH, –C=O, and –C–O suggests the successful formation of ERGO due to the electrochemical deoxygenation of GO, which corroborates the Raman analysis.

Figure 1D depicts a characteristic XRD peak of GO at 2θ = 9.98 (interplanar spacing = 0.843 nm) corresponding to 001 reflections. Two characteristic peaks of ERGO at 2θ = 21.15 (interplanar spacing = 0.413 nm) and 2θ = 29.65 (*d*-spacing = 0.343 nm) for the reflection of (020) and (200), respectively, confirm the successful formation of ERGO from GO.

**Figure 2:**
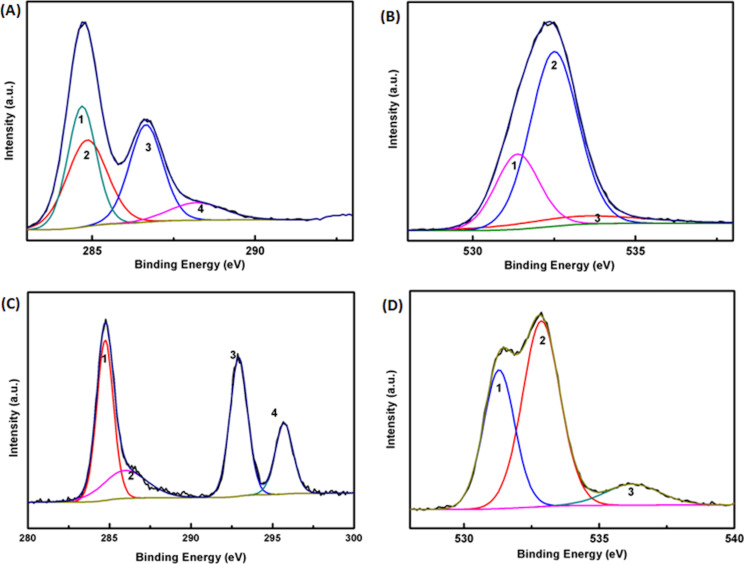
Deconvoluted XPS core-level spectrum of (A) C 1s, (B) O 1s for GO, (C) C 1s, and (D) O 1s for ERGO samples, respectively.

Figure 2 represents the deconvoluted C 1s and O 1s XPS spectra of GO (Figure 2A and Figure 2B) and modified ERGO (Figure 2C and Figure 2D) electrodes. An asymmetric peak centered on ≈284.8 eV appeared due to the graphitic nature of GO and ERGO (Figure 2A and Figure 2B). Four different carbon types are observed from the deconvolution of the peaks shown in Figure 2A. They show an increase in binding energies evidencing the presence of C–OH, C–C, C–O–C, and C=O bonds in GO. The O 1s spectra of synthesized GO can be deconvoluted into three peaks, corresponding to contributions from carbonyl and carboxyl-type oxygen (531.4 eV), C–OH type (532.5 eV), and hydroxyl (533.6 eV). The intensity of the peaks is significantly reduced in RGO samples (Figure 2C and Figure 2D) compared to pristine GO, indicating considerable deoxygenation. The C 1s spectra of RGO (Figure 2C) can also be deconvoluted into four peaks at 284.7, 285.96, 292.8, and 295.7 eV. However, the relatively intense doublet appeared at 292.8 ± 0.1 eV and beyond 295 eV every time we performed the scan. Peaks in the range of 290 eV in these types of materials are mainly due to aromatic π–π^*^ transitions. However, considering the intensity of the peak and our repeated measurements, we believe that the presence of a well-defined deconvoluted doublet peak beyond 290 eV corresponds to K 2p_3/2_ and K 2p_1/2_, which may have resulted from the contribution coming from the potassium salt present in the buffer during electrochemical conversion. The deconvoluted analysis of the peaks and the relative atomic percentages of GO and RGO are summarized in Supporting Information File 1, Table S2.

**Figure 3:**
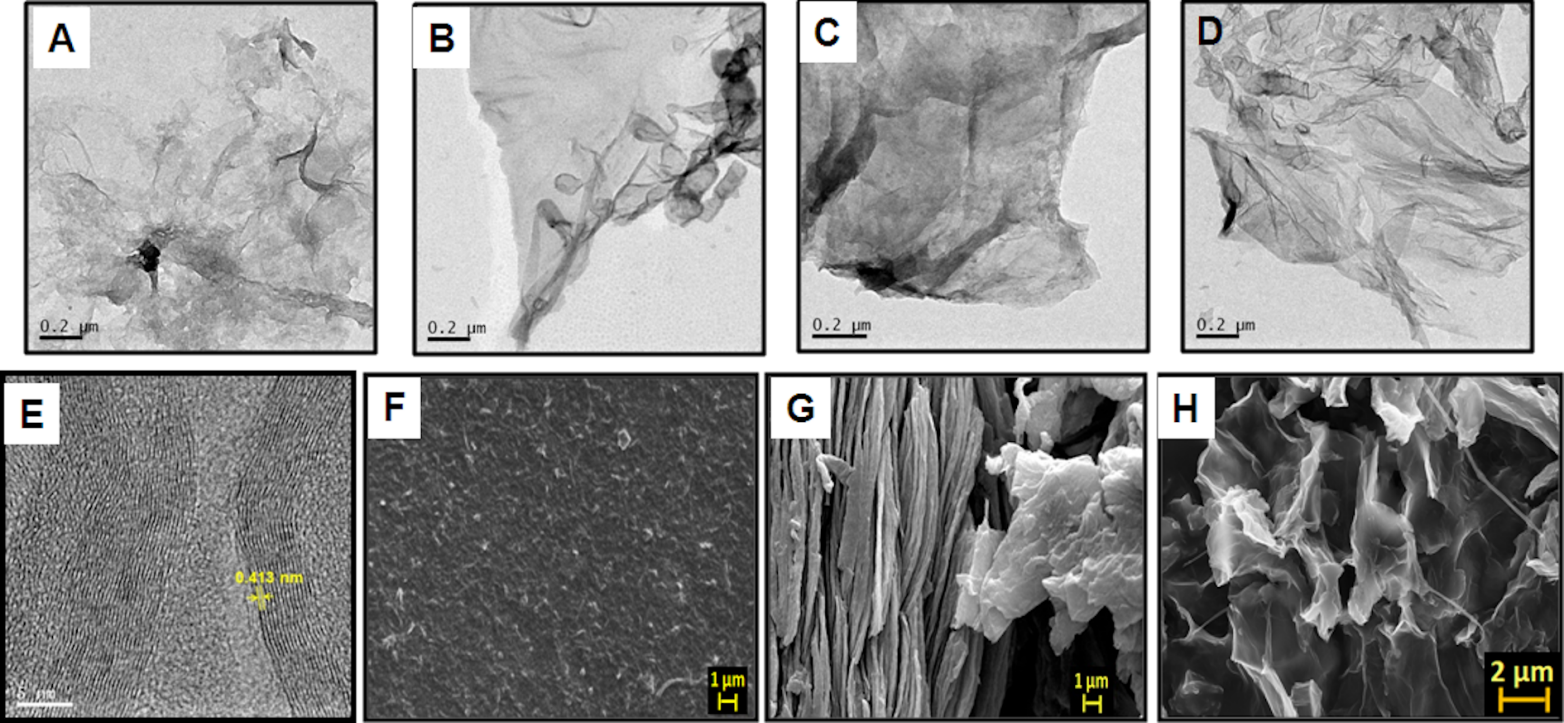
(A) TEM images of as-synthesized GO, ERGO synthesized in different electrolytes: (B) PBS pH 4.5, (C) pH 7, and (D) pH 9.6. (E) HRTEM image of ERGO in PBS pH 4.5. (F) SEM micrographs of as-synthesized GO, (G) ERGO in PBS pH 4.5 and (H) FESEM of ERGO in PBS pH 4.5 at different magnifications.

Figure 3A–D depicts TEM micrographs of as-prepared GO and synthesized ERGO at different pH values, indicating that the intensity of the electrons is attenuated by the platelets of graphene sheets with varying transparencies due to thickness variation [26,31]. Dark areas of the micrograph suggest thick stacking layers of GO and/or RGO with intercalated oxygen-containing functional groups. A few layers of graphene sheet in ERGO (in PBS, pH 4.5) have areas with higher transparency due to the exfoliation of stacking layers of GO. This suggests an increased surface area due to delamination of graphene layers (thickness of about one to a few layers) by electrochemical reduction. The high-resolution TEM of ERGO shows a *d*-spacing of 0.413 nm (Figure 3E), indicating a reduced graphite nature of GO. This confirms that the oxygen functional groups were removed from the graphene layers by electrochemical reduction of GO, decreasing the interspacing distance between graphene layers which facilitates electron transport. Thus, the conductivity of ERGO was enhanced compared to that of GO. The SEM micrograph of ERGO (Figure 3G) also shows graphene sheet exfoliated layers compared to GO (Figure 3F). The FESEM image also depicts the flaked nanostructure of RGO (Figure 3H).

### Electrochemical characterization of the modified electrode

**Figure 4:**
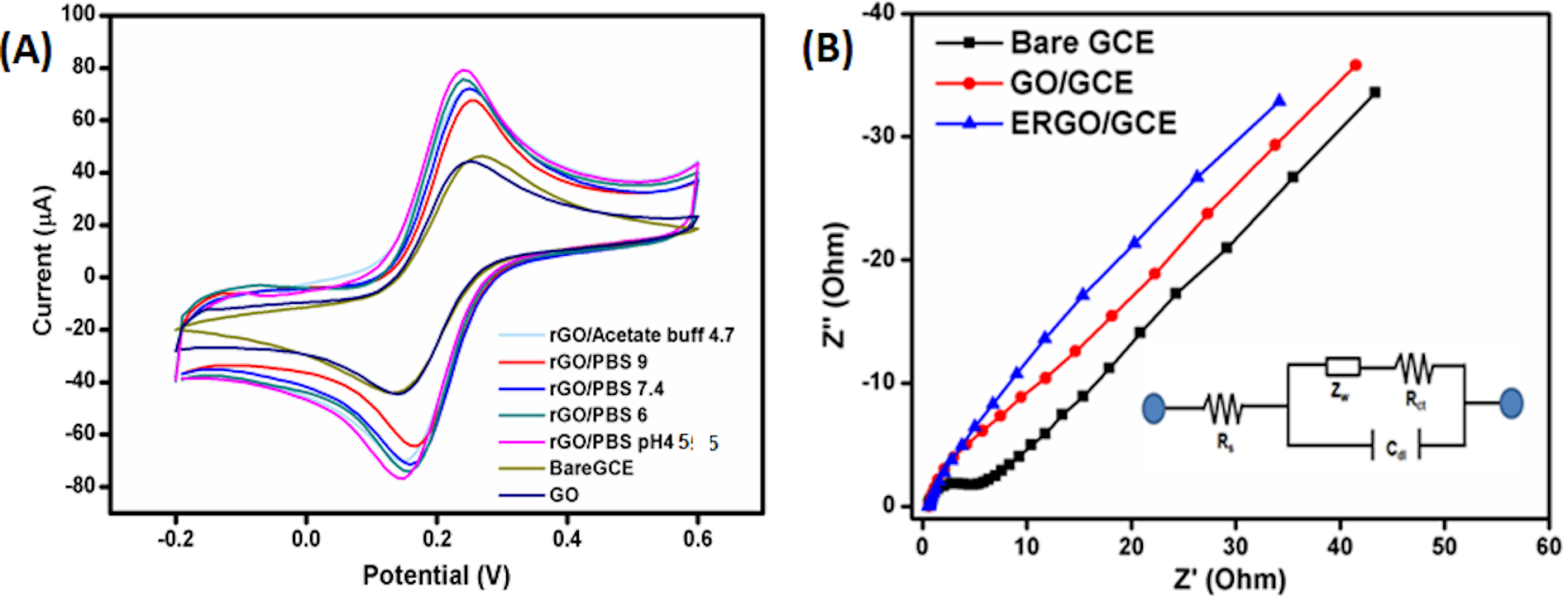
(A) Cyclic voltammograms of GO and ERGO using different electrolytic buffers: Acetate buffer pH 4.7, PBS pH 4.6, PBS pH 6, PBS pH 7.4, PBS pH 9. (B) Nyquist plot of bare GCE, GO/GCE, and ERGO/GCE in the presence of 1 mM [Fe(CN)_6_]^4−/3−^ containing 0.1 M KCl.

The electronic properties of graphene materials depend on the number of layers and the distance between the layers, which can be changed by a variation of the synthesis protocol to achieve a higher electroactive surface area and electrical conductivity. Figure 4A displays a higher oxidation/reduction peak current of Fe^2+/3+^ redox couple for the synthesized ERGO in PBS pH 4.5. It forms the highest electroactive surface area compared to other electrolytic buffers and pH values to prepare ERGO/GCE. To confirm the increase in the electroactive surface area of ERGO/GCE in comparison to bare GCE, CV was performed at different scan rates (10–300 mV/s) in 1.0 mM K_3_Fe(CN)_6_ as a redox probe (Supporting Information File 1, Figure S1). The electroactive surface areas were calculated according to Randles–Sevcik equation (Equation 1) [28,32]:


[1] $$I_{\text{p}}=\left(2.69\times 10^{5}\right)n^{3/2}A_{\text{c}}D_{\text{r}}^{\frac{1}{2}}v^{\frac{1}{2}}C_{0},$$

where *I*_p_ is the peak current (A), ν is the scan rate (V s^−1^), *n* is the number of electrons transferred (*n* = 1), *A*_c_ is the electrode active area (cm^2^), *D*_r_ is the diffusion coefficient (7.6 × 10^−6^ cm^2^·s^−1^), and *C*_0_ is the concentration of K_3_Fe(CN)_6_ (mol·cm^−3^). From the slope of the plot of *I*_p_ vs ν^1/2^, the effective surface area for bare GCE and ERGO/GCE was calculated to be 0.0707 and 0.121 cm^2^, respectively, which indicates that the effective electroactive surface area of ERGO has been improved by ≈71.14% due to exfoliation of graphene sheets.

Electrochemical impedance spectroscopy was performed to investigate the electron transfer capability of ERGO (Figure 4B). Supporting Information File 1, Table S3 depicts the values of charge-transfer resistance (*R*_ct_), capacitance (*C*_dl_), and Warburg impedance (*W*) of bare GCE, GO/GCE, and ERGO/GCE. The Nyquist plot of the bare GCE electrode depicts a semicircle with *R*_ct_ of 4.692 Ω. A nearly straight line for ERGO with a negligible *R*_ct_ (1.618 Ω) value suggests opened porous microstructures of ERGO, which makes the graphene sheets more accessible to the electrolyte. It also facilitates electron transfer and diffusion of ions during the electrochemical process [28,34].

### Electrochemical behavior of parathion at modified nanosensors

**Figure 5:**
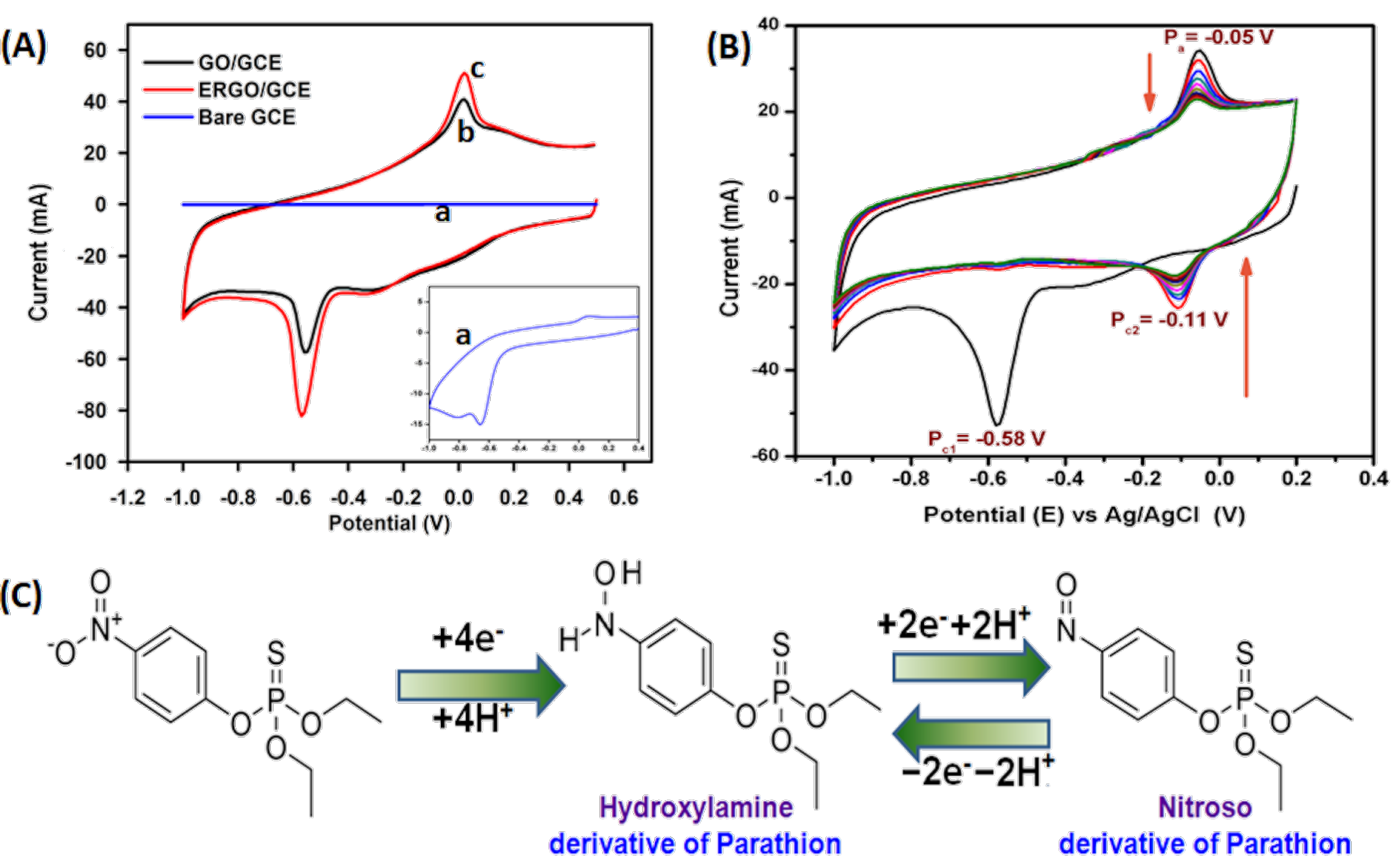
(A) Cyclic voltammograms of PT (10 mM) with bare GCE (a), GO/GCE (b), and ERGO/GCE (c). (B) Electrochemical behavior of PT at ERGO/GCE. (C) Schematic diagram of the proposed electrochemical reaction of parathion at ERGO/GCE.

Figure 5A depicts the CVs (first cycle) of bare GCE, GO/GCE, and ERGO/GCE in PBS (0.05 M, pH 7) in 10 μM PT. The CV of PT on bare GCE (inset of Figure 5A), shows a reduction peak at −0.65V and a little anodic peak due to autocatalysis of PT. A robust cathodic peak at −0.56 V and an anodic peak at +0.015 V were mainly observed on GO/GCE due to the absorption of PT through π stacking interaction between aromatic moieties of GO and the benzene ring of PT. In comparison, the highest cathodic/anodic peak was obtained at −0.58 and −0.05 V, respectively, for the electro-reduction/oxidation of PT on ERGO/GCE. The oxidation/reduction potentials of PT on ERGO/GCE were shifted to less positive values, effectively inhibiting the surface fouling caused by the reaction products, making ERGO-modified GCE more suitable for determining PT. The electrocatalytic ability of PT (10 µM) on the modified ERGO/GCE was investigated in PBS (pH 7) (Figure 5B) in the potential range from +0.2 to −1.0 V with a scan rate of 100 mV·s^−1^ and compared with the control group (bare GCE and GO/GCE). It is in good agreement with the literature reports that a sharp cathodic peak (*E*_pc1_) at −0.58 V was observed in the first cycle due to the reduction of the nitro group of PT (NO_2_–PT) to form its hydroxylamine derivatives (NHOH–PT) involving a four electron-transfer process as shown in Figure 5C [16–18,35]. An anodic peak appeared at −0.05 V in the backward segment of the first cycle, which is related to the oxidation of NHOH–PT to a nitroso group (NO–PT). This reversible two-electron-transfer process further generated a reduction peak (*E*_pc1_) at −0.11 V during the second potential scan of CV (Figure 5C). Nitroaromatic OPs such as parathion, methyl parathion, ethyl parathion, and fenitrothion, paraoxon exhibit this kind of electrocatalytic behavior, which is consistent with previous reports [21,23–24]. In this study, we chose the irreversible reduction peak of PT (NO_2_–PT to NHOH–PT) of the first cycle due to its suitability for important measurements in nanosensor applications.

**Figure 6:**
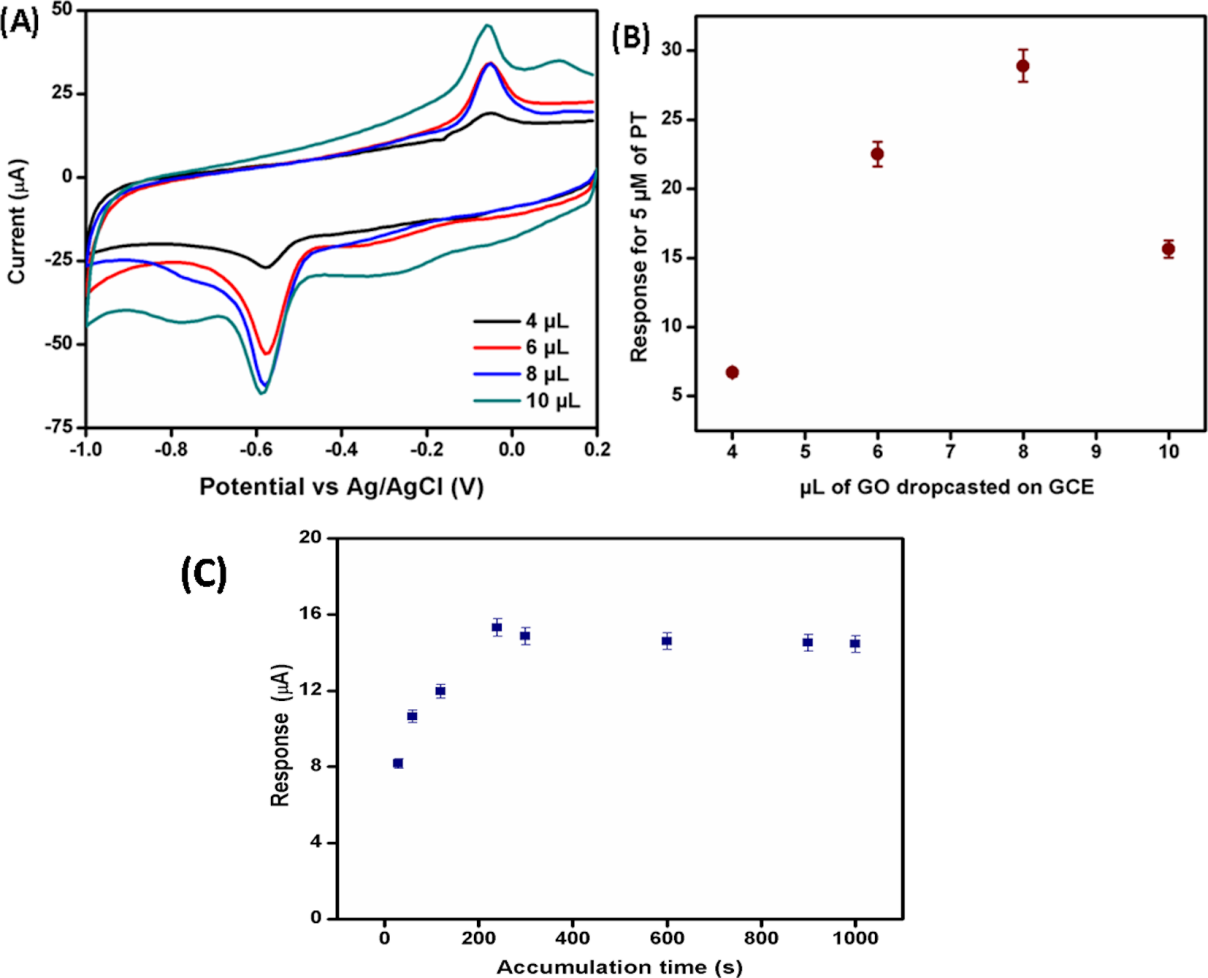
(A) Cyclic voltammograms of 10 μM PT in PBS, pH 7, with different amounts of GO deposited on bare GCE for the preparation of modified ERGO/GCE. (B) Peak current of cyclic voltammetry using 10 μM PT with different amounts of GO deposited on bare GCE for preparation of modified ERGO/GCE. (C) Peak response of 10 μM PT with variation of the accumulation time before voltammetric measurements.

The amount of exfoliated GO dispersed on bare GCE is vital in optimizing the sensing matrix. Figure 6A depicts the CVs using a variation of deposited GO on bare GCE to prepare ERGO/GCE to measure the reduction and oxidation peak current for 10 μM PT in PBS, pH 7. Figure 6B shows that the highest reduction peak for 10 μM PT was obtained using 8 μL of GO to prepare modified ERGO/GCE. As the autocatalytic response for the electrochemical oxidation/reduction process is an absorption process, the accumulation time is another vital parameter to achieve the highest response for monitoring the amount of parathion residue in samples [35–36]. It has been shown in Figure 6 that as the immersion time of the modified electrode in a PT solution increased, the accumulation of PT on the electrode surface also enhanced. It was found that the highest peak current for 10 μM PT was obtained after immersion for 240 s in the PT solution. A further increase in the accumulation time was unaffected as the active area of the electrode surface was saturated **(**Figure 6C).

### Effect of scan rate and pH values on the electrolyte

**Figure 7:**
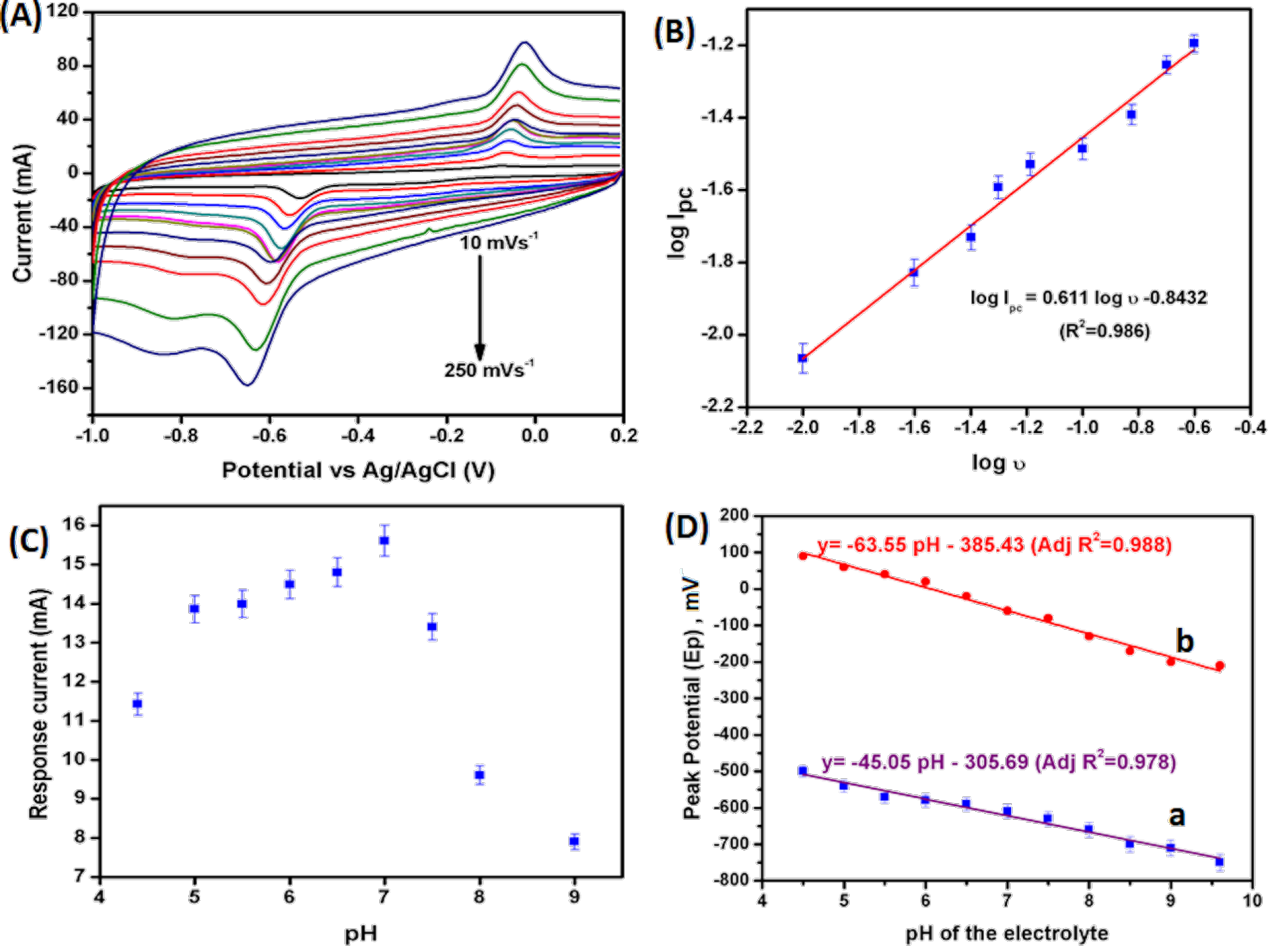
(A) Cyclic voltammograms of ERGO/GCE under different scanning rates (10, 25, 40, 50, 65, 80, 100, 125, 150, 200, 250 mV·s^−1^) in PBS, pH 7, containing 10 μM PT. (B) Plot of the logarithm value of the reduction peak current as a function of the scanning rate (log *I*_pc_ as a function of log ν). (C) Plot of the reduction peak current of PT as a function of electrolyte pH. (D) Plot for the reduction (a) and oxidation (b) peak potential of PT (40 μM PT) as a function of the electrochemical cell pH, scan rate: 100 mV·s^−1^.

The effect of scan rate on the reduction of PT at ERGO/GCE was investigated by applying different scan rates from 10 to 250 mV·s^−1^ (Figure 7A). The linear peak current increase with the scan rate suggests a surface-confined diffusion-controlled electrocatalytic process [21]. The slope of log *I*_pc_ as a function of log ν is 0.611 (>0.5), which confirms an adsorption-based reduction of PT on the modified electrode surface (Figure 7B). The reduction peak potential was shifted towards a more negative potential by increasing the scan rate. A linear equation of *E*_p_ as a function of log ν was represented as *E*_p_ = −0.088log ν − 0.694, with a correlation coefficient of (R²) 0.992. From the Laviron’s equation (Equation 2) for an irreversible reaction, *E*_p_ could be represented as


[2] $$E_{\text{p}}=E{{}^{\circ}}^{\prime }\pm \left(\frac{2.303RT}{\propto nF}\right)\log\left(\frac{RTk{}^{\circ}}{\propto nF}\right)\pm \left(\frac{2.303RT}{\propto nF}\right)\log\nu ,$$

where ∝ is the transfer coefficient; *n* is the number of electron transfers; and *R*, *T*, *F* represent constants (*R* = 8.314 J·K^−1^, *T* = 298 K, *F* = 96480 C·mol ^−1^). The standard redox potential (*E°*’) was found to be −0.523 V from the linear plot of *E*_p_ as a function of ν (*E*_p_ = −0.908ν − 0.523), at a scan rate 0 Vs^−1^. The standard heterogeneous rate constant (*k*°) for electrocatalysis of PT was 38.81 s^−1^. The value of ∝*n* was calculated to be 0.672, and the *n* value was found to be 0.954 (i.e., one-electron transfer process [37]).

The protonation reaction influences the electrochemical reaction. Figure 7C shows the effect of pH on the electroreduction of PT (40 μM) by varying the pH values of PBS from 4.6 to 9. The irreversible reduction potential of PT was shifted towards a more negative potential as the pH values of the electrolyte varied from 4.5 to 9 (Figure 7D). The slope of the reduction peak (*E*_pc_) and oxidation peak (*E*_pa_) potential of PT as a function of pH is near −59 mV, which suggests that the same number of e^−^ and H^+^ is involved in the reaction [38–39].

### Optimization of square-wave voltammetry parameters

**Figure 8:**
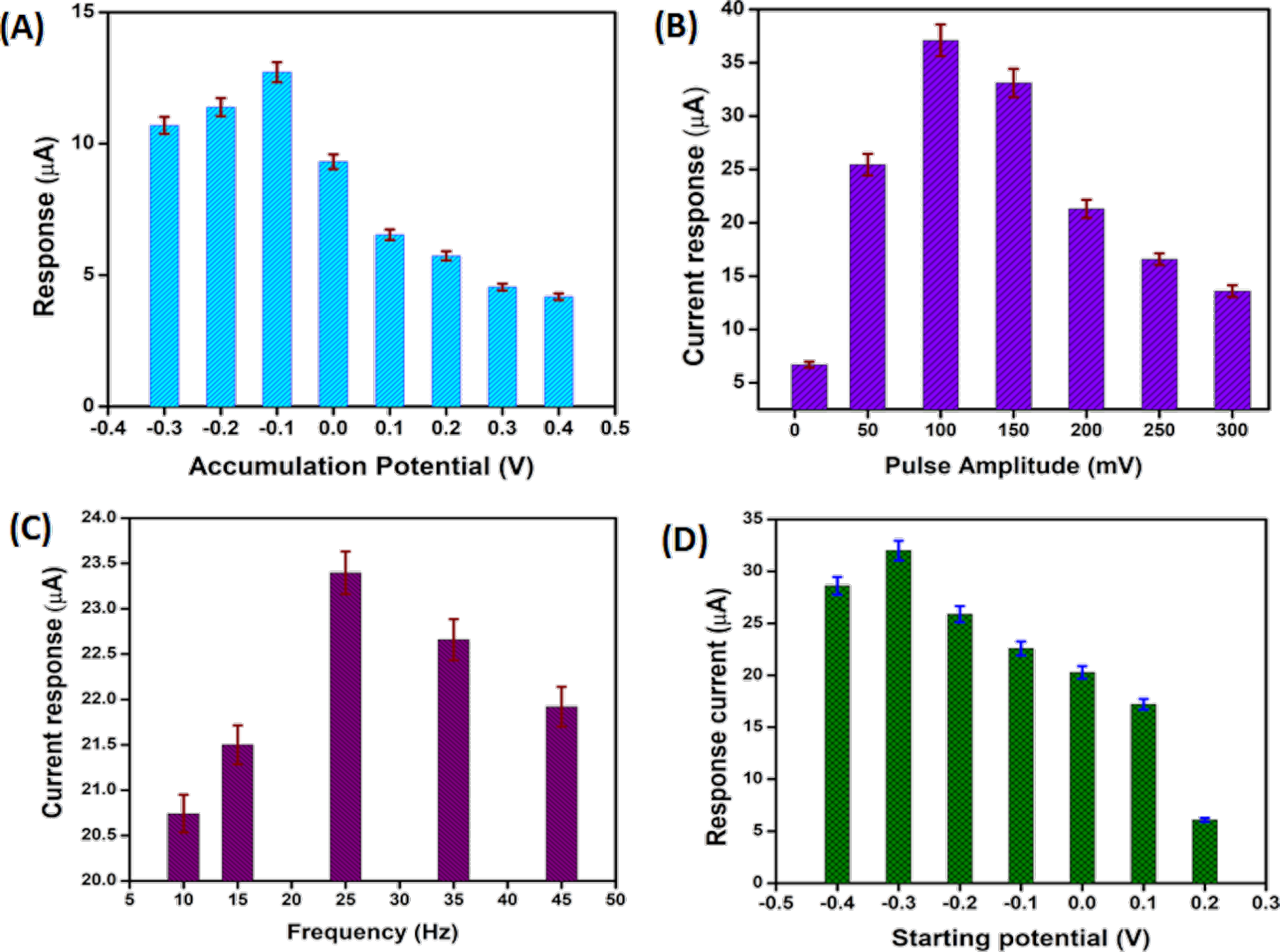
Relationship of stripping peak current in SWV measurements of 10 μM PT as a function of accumulation potential (A), starting potential of scan (B), frequency (C), and pulse amplitude (D). The optimized parameters for PT detection in SWV are: pulse amplitude: 100 mV, starting potential: 0.3 V, frequency: 25 Hz, accumulation potential: −0.1 V, and accumulation time: 240 s.

Square-wave voltammetry analysis is more accurate compared to an electrochemical method such as cyclic voltammetry and differential pulse voltammetry. It can minimize background current to obtain an intense, sharp, and well-defined peak of the targeted analyte at a particular potential. To obtain the maximum peak current, the parameters of SWV were optimized using 10 μM PT in PBS (pH 7). The variation of reduction peak current with accumulation potential (A), starting potential of scan (B), frequency (C), and pulse amplitude (D) are shown Figure 8A–D.

### Analytical performance and selectivity of the proposed nanosensor

**Figure 9:**
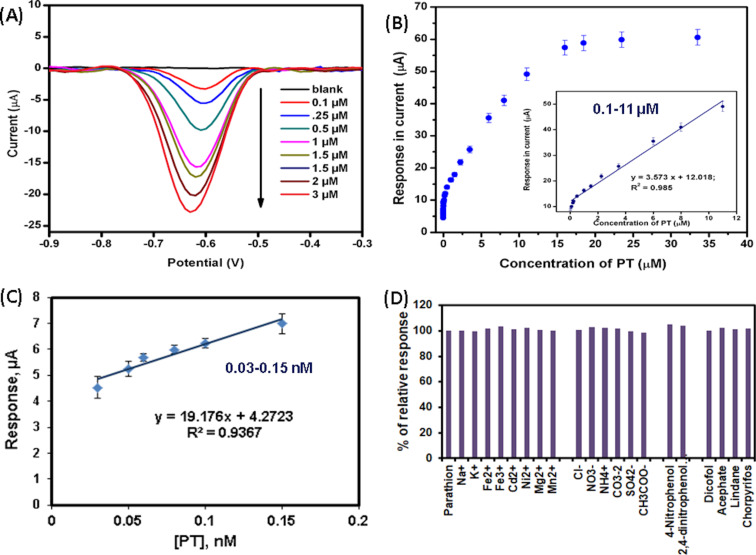
(A) SWV response of ERGO/GCE electrode in electrolytes (PBS, 0.05 M, pH 7) with different concentrations of PT ranging from 0.1 to 3 μM. (B) Calibration plot of peak current as a function of PT concentration for a wide range (i.e., 3 × 10^−5^ to 11 µM, inset: linear regression curve for 0.1 to 11 µM) and (C) 0.03–0.15 nM. (D) Selectivity studies of PT (10 μM) detection with probable interfering substances such as Na^+^, K^+^, Fe^2+^, Fe^3+^, Cd^2+^, Ni^2+^, Mg^2+^, Mn^2+^, NH_4_^+^, Cl^−^, NO_3_^−^, CO_3_^2−^, SO_4_^−^, CH_3_COO^−^, 4-nitrophenol, 2,4-dinitrophenol, acephate, chlorpyrifos, dicofol, and lindane, respectively using SWV and keeping all other parameters constant. Experimental conditions: pulse amplitude: 100 mV, SWV frequency: 25 Hz, starting potential: 0.3 V, accumulation potential: −0.1 V, and accumulation time: 240 s.

Figure 9A represents SWV curves obtained from the ERGO modified electrode for sequential additions of PT into phosphate buffer (pH 7). A sharp increase in the reduction peak current was observed for each addition after dipping the electrode into a particular solution for 240 s at an applied potential of −0.1 V (i.e., deposition potential). The peak was shifted to a negative potential as the concentration of PT enhanced, indicating a diffusion-controlled process [40]. The concentration-dependent linear plot depicts good linearity (Figure 9B, Figure 9C) with a calibration equation of *I*_p_ (μA) = 3.5735 [PT] + 12.018 (*R*² = 0.9871) for the range of 0.1–11 μM, *I*_p_ (μA) = 0.2916 [PT] + 3.7526 (*R*² = 0.9936) for 3–15 nM, *I*_p_ (μA) = 19.176 [PT] + 4.2723 (*R*² = 0.9367) for 0.03–0.15 nM. The corresponding sensitivity was found to be 50.5 μA·μM^−1^·cm^−2^ with a wide linear range for quantification of PT. The limit of detection (LOD = [(3 * standard deviation of blank)/slope of the lowest range of linear curve (i.e., 0.03–0.15 nM)] and limit of quantification (LOQ = [(10 * SD of blank)/slope]) were calculated as 10.9 pM and 36.5 pM, respectively, from the lower calibration equation [39,41].

The selectivity of the proposed ERGO/GCE modified nanosensor (Figure 9D) was investigated in the presence of other possible substances in water and soil samples. Square-wave voltammetry measurements were performed in PBS (50 mM, pH 7.0) containing 10 μM PT along with some inorganic ions (e.g., Na^+^, K^+^, Fe^2+^, Fe^3+^, Cd^2+^, Ni^2+^, Mg^2+^, Mn^2+^, NH_4_^+^, Cl^−^, NO_3_^−^, CO_3_^2−^, SO_4_^−^, CH_3_COO^-^), nitroaromatic compounds (e.g., 4-nitrophenol, 2,4-dinitrophenol), and other pesticides, such as acephate, chlorpyrifos, dicofol, lindane. As shown in Figure 9D, the modified electrode showed almost the same peak current when PT coexists with other substances. This indicates that the added substances have no significant effect on PT sensing in environmental samples. Other interfering OP (acephate, chlorpyrifos) and organochloride (dicofol, lindane) pesticides also did not significantly affect the response current of PT reduction as they have different redox potential and adsorption potential on the modified electrode surface.

Reproducibility, repeatability, and stability are essential parameters for practical applications of electrochemical nanosensors. Inter-assay measurements of 10 μM PT using five independent ERGO/GCE were performed, and a 3.4% relative standard deviation (RSD) was obtained for five replicate scans, indicating good reproducibility of the proposed nanosensor. Similarly, a single modified electrode exhibits good repeatability with an RSD of 1.81% for five repeated measurements performed in PBS (50 mM, pH 7.0) containing 10 μM PT.

The analytical performance of the ERGO/GCE, such as detection limit and linear range, are compared with previously reported modified electrodes for the detection of PT (Table 2) [16–18,36,42–44]. The proposed electrode showed better stability, sensitivity, and the lowest detection limit in comparison to previous reports [16–18,36,42–44]. As ERGO showed thermal and mechanical stability, ERGO/GCE could be a suitable electrode material for rapid screening of PT in actual samples.

**Table 2:** Experimental sample table for a comparative analytical performance of the proposed nanosensor with the reported nonenzymatic nanosensor.

Modified electrode	Method	Molecule	Linear range (μM)	LOD (nM)	pH	Samples	Ref

NanoTiO_2_-SAM/GCE	DPV	PT	0.05–10	10	PBS 5	cucumber, cabbage	[17]
NanoAg/Naf ion/GCE	DPV	PT	0.103–0.62	80	BR buffer, pH 2.56	water	[16]
		MP	0.300–1.444	0.0874			
ZrO_2_/MAS/Au	SWV	PT	0.017–3.4	2.8	pH 6, 0.1 M KCl	vegetables, water	[43]
SPAN(sulfonated Pani)/GCE	DPV	PT	0.01–10	1.5	BR buffer 2.5	urine sample	[42]
ordered mesoporous carbon/GCE	DPV	PT	0.015–0.5	3.4	PBS 6	–	[44]
NiO-SPE	DPV	PT	0.1–30	24	0.05 M BR buffer, pH 6.0	urine, tomato	[18]
Al-doped mesoporous cellular foam (Al-MCF)	SWV	PT	0.01–1 mg/L	17.16	0.1 M KCl, pH 6.0	cabbage	[36]
ERGO/GCE	SWV	PT	3 × 10^−5^–11	10.9 × 10^−3^	PBS, pH 7	groundwater, soil, tomato, rice	present work

To determine the storage stability, the electrocatalytic response of 10 μM PT was monitored in seven-day intervals for the first two months, and it retained about 96.17 ± 0.2% of its initial response. It was shown a consistent response to PT sensing during two months of storage. After that, the response was measured in intervals of 10 days, and 90.53 ± 0.3% of the initial response was retained after six months. This indicates good stability of the modified electrode at room temperature (Supporting Information File 1, Figure S2A). The feasibility of the proposed robust sensing platform was demonstrated by quantifying environmental samples such as groundwater and a soil sample from an agricultural land. Food (e.g., boiled rice) and vegetable (e.g., tomato collected from local market) samples were also analysed. As the concentration of PT in the collected samples was negligible, a specific amount of PT was spiked from the standard PT solution (1 mM). Supporting Information File 1, Figure S2B depicts the SWV response of groundwater spiked with 1.5, 2.5, and 5 μM PT, and detailed experimental results are shown in Table 3. The amount of spiked [PT] was monitored by the SWV response, and the results were validated using standard UV results. The UV spectra with increasing PT concentration (1–35 µM) are shown in Supporting Information File 1, Figure S3A. The concentration of PT in real samples was further calculated from the standard calibration curve obtained from UV spectra at 273 nm (Supporting Information File 1, Figure S3B). The quantitative spiked recoveries of PT ranged from 97.0–102.4%, with an RSD of 0.998–1.62%. In addition, the proposed method also depicts satisfactory relative error (1.53–3.96%) with standard absorption results for the quantification of PT in environmental samples.

**Table 3:** Experimental sample table for recovery studies of spiked PT in actual samples.

Real samples	Added (μM)	Detected (μM)	Detected by UV–vis	Recovery (%)	Relative error (%)	RSD (%)

ground water	1.5	1.46	1.52	97.33	3.947	0.998
	2.5	2.48	2.52	99.20	1.587	1.518
	5	5.12	5.2	102.4	1.538	1.369
soil	1	0.97	1.01	97.00	3.961	1.620
	3	2.95	3.07	98.33	3.909	1.114
	5	4.96	5.04	99.20	1.587	1.240
tomato	1	0.96	0.99	96.00	3.030	1.120
	3	2.9	3.04	96.67	4.605	0.992
	7	7.07	7.18	101.00	1.532	1.060
rice	0.5	0.51	0.58	102.00	12.06	1.119
	5	5.12	5.16	102.40	0.775	0.991
	10	10.11	10.25	101.10	1.366	1.230

## Conclusion

A newly developed inexpensive and environmentally friendly techique, using an interference-free nonenzymatic approach was developed to fabricate a nonenzymatic electrochemical nanosensor based on ERGO for rapid detection of PT. The electrochemical parameters were optimized to achieve the highest performance of the ERGO-modified electrode, and the structure was characterized by Raman, XRD, XPS, TEM, FESEM, and EIS techniques. Square-wave voltammetry was performed to achieve excellent nanosensor performance, such as higher sensitivity, low detection limit (10.9 pM), linear response range (3 × 10^−11^–11 × 10^−6^ M), and fast response time. The proposed ERGO/GCE nanosensor exhibits excellent electrocatalytic activity, long-term storage stability, reproducibility, repeatability, low-cost fabrication, and strong anti-interference ability to quantify PT residues in real samples. The low RSD value (0.998–1.62%) and relative error (1.9–3.9%) obtained from UV data confirmed the accuracy of this method, showing that the electrochemical nanosensor has good reliability for PT detection in real samples. It can be concluded that the feasible nonenzymatic electrochemical nanosensor could be a good alternative for on-site monitoring of PT usage in agricultural fields. The robust and straightforward electrochemical-sensing platform could also be a promising path for selective and sensitive analysis of other pesticides and environmental pollutants based on electrocatalytic activity.

## Supporting Information

Table S1: Chemical sample table indicating corresponding CAS, supplier and other details. Table S2: Experimental sample table for composition analysis using binding energies of GO and RGO by XPS. Table S3: Experimental sample table for the modified glassy carbon electrode electrochemical characteristics. Figure S1: CV of ERGO/GCE at different scan rates (10–300 mV/s) in 1.0 mM K_3_Fe(CN)_6_ solution with 1 M KCl. Figure S2: (A) Storage stability of the proposed sensing matrix (ERGO/GCE), (B) SWV of PT (1.5, 2.5, 5 μM) added in groundwater. Figure S3: (A) Absorption spectra of parathion and (B) corresponding calibration plot.

File 1 Additional figures and tables.
