# Supplementary material for: A nonenzymatic reduced graphene oxide-based nanosensor for parathion
Source: Beilstein J Nanotechnol. 2022 Jul 28;13:730–44. doi: 10.3762/bjnano.13.65 (PMC9344548; doi:10.3762/bjnano.13.65)
Supplement: File 1 — Additional figures and tables. [file Beilstein_J_Nanotechnol-13-730-s001.pdf]

BEILSTEIN JOURNAL OF NANOTECHNOLOGY

Supporting Information

for

A nonenzymatic reduced graphene oxide-based nanosensor for parathion

Sarani Sen, Anurag Roy, Ambarish Sanyal and Parukuttyamma Sujatha Devi

Beilstein J. Nanotechnol. **2022,** *13,* 730–744. doi:10.3762/bjnano.13.65

Additional figures and tables

License and Terms: This is a supporting information file under the terms of the Creative Commons Attribution License (https://creativecommons.org/licenses/by/4.0). Please note that the reuse, redistribution and reproduction in particular requires that the author(s) and source are credited and that individual graphics may be subject to special legal provisions.
The license is subject to the *Beilstein Journal of Nanotechnology* terms and conditions: (https://www.beilstein-journals.org/bjnano/terms)

Table S1: Chemical sample table indicating corresponding CAS, supplier and other details.

**Component**	**CAS Number**	**Supplier**	**Features**
Parathion PESTANAL	56-38-2	Merck, India<br>(45607)	Molecular Weight: 291.26<br>Analytical Standard
Graphite Powder	7782-42-5	Thermo Fisher Scientific -<br>Fisher Chemicals<br>(G090060)	Molecular Weight (g/mol) 12.011<br>99% Pure
Sulfuric Acid	7664-93-9	Thermo Fisher Scientific -<br>Qualigens<br>(Q37365)	N/50 Solution
Nitric Acid	7697-37-2	Thermo Fisher Scientific -<br>Qualigens<br>(Q29195)	1.42 (70%)<br>Product Grade: SQ, ER
Hydrogen Peroxide	7722-84-1	Thermo Fisher Scientific -<br>Qualigens<br>(Q15465)	30%<br>Product Grade: SQ, ER
Sodium Dihydrogen Phosphate<br>Dihydrate	13472-35-0	Merck, India<br>SKU: 1063420250	Molecular Weight:<br>156.01<br>analysis EMSURE®
Sodium Acetate Buffer	126-96-5	Merck, India<br>SKU: S7899	pH 5.2 ± 0.1 (25 °C), for molecular biology, 3 M, 0.2 μm filtered

Table S2: Experimental sample table for composition analysis using binding energies of GO and RGO obtained by XPS.

**Sample**	**C 1s**				**O 1s**		
	C–OH	C–C	C–O–C	C=O	–C=O	C–OH	–OH
GO	284.69 eV (30.9 %)	284.84 eV (31.2 %)	286.65 eV (29.2 %)	288.21 eV (8.6 %)	531.38 eV (25.9 %)	532.51 eV (65.7 %)	533.21 eV (8.4 %)
ERGO	284.73 eV (35.1 %)	285.96 eV (15.9 %)	292.92 eV (32.3 %)		531.30 eV (34.9 %)	532.86 eV (55.9 %)	536.22 eV (9.2 %)

Table S3: Experimental sample table for modified glassy carbon electrode electrochemical characteristics.

**Sample**	R_s **(Ω)**	R_{ct} **(Ω)**	**Cdl (F)**	**W [Ω·sqrt(Hz)]$^{-1}$**
Bare GCE	0.0872	4.692	1.96E−04	0.06072
GO/GCE	0.7326	6.938	1.06E−02	0.06176
ERGO/GCE	0.6013	1.618	1.96E−02	0.08136

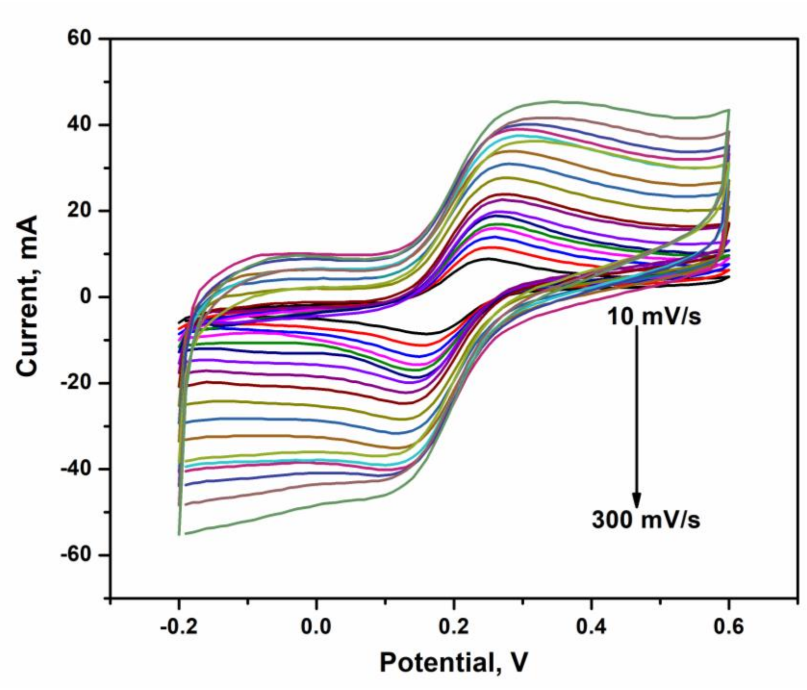

Figure S1: Cyclic voltammograms of ERGO/GCE at different scan rates (10–300 mV/s) in 1.0 mM $K_3Fe(CN)_6$ solution with 1 M KCl.

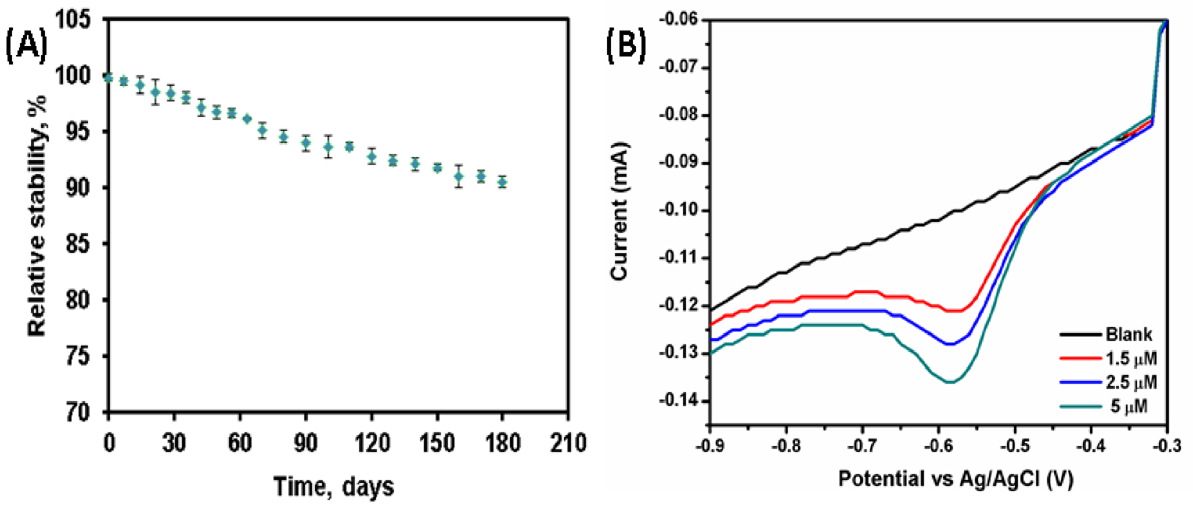

Figure S2: (A) Storage stability of the proposed sensing matrix (ERGO/GCE). (B) SWV of PT (1.5, 2.5, 5 μM) added in groundwater.

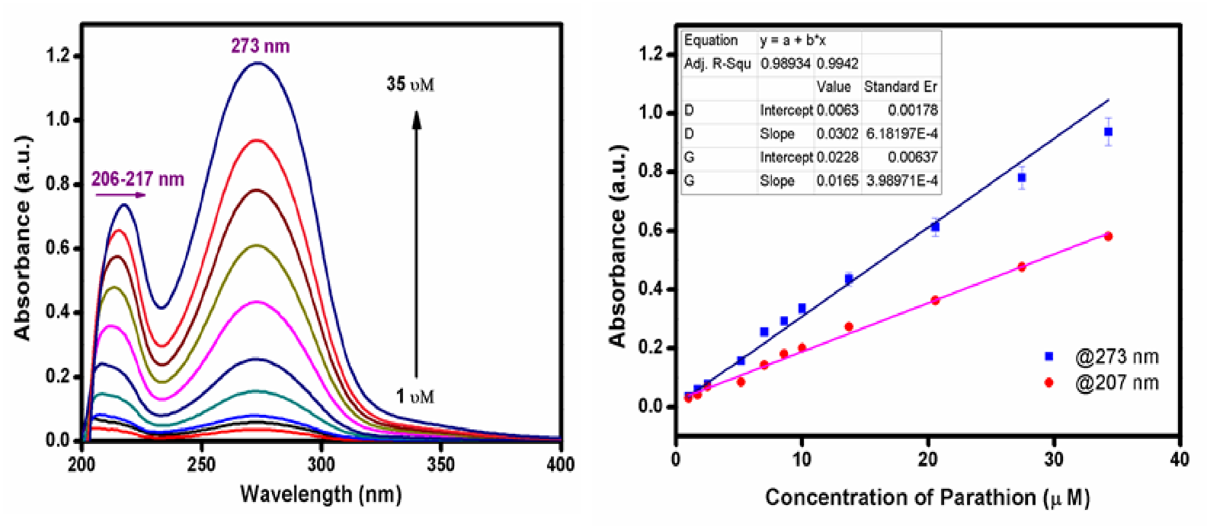

Figure S3: (A) Absorption spectra of parathion and (B) corresponding calibration plot.
